# Road Blocks or Building Blocks? A Qualitative Study on Challenges and Resilience in Romantic Relationships of Youth Exposed to Family Violence

**DOI:** 10.1007/s40653-023-00592-4

**Published:** 2023-12-08

**Authors:** M. K. M. Lünnemann, F. C. P. Van der Horst, D. Van de Bongardt, M. Steketee

**Affiliations:** 1 Erasmus University Rotterdam, PO Box 1738, Rotterdam, 3000 DR the Netherlands; 2 Verwey-Jonker Institute, Kromme Nieuwegracht 6, Utrecht, 3512 HG The Netherlands

**Keywords:** Romantic Relationships, Youth, Family Violence, Challenges, Resilience

## Abstract

Romantic relationships are an important part of many people’s lives and at least partly shaped by experiences during childhood. Youth exposed to family violence during childhood are more likely to experience difficulties in their later romantic relationships. However, a more holistic perspective on the romantic relationships of youth with a history of family violence is lacking. Using both theoretical and inductive thematic analysis, this qualitative study explored challenges as well as positive experiences within romantic relationships of youth exposed to family violence during childhood. In-depth individual interviews were conducted with 18 youth aged between 16 and 20 years, who were reported to child protection services. The narratives reflected that youth experienced challenges related to support, connection, trust, boundary setting, emotion regulation and conflict resolution. Furthermore, family violence during childhood seemed to be important in the emergence of these challenges, consistent with theoretical mechanisms described in observational learning theory and attachment theory. However, youth also described positive experiences in their romantic relationships and demonstrated an ability to learn from others (e.g., their current romantic partner) how to communicate effectively or solve problems. Therefore, with the right social or professional support, at-risk youth may be able to overcome these challenges.

## Introduction

Relationships, including romantic relationships, are an essential part of our species history (cf. Hrdy, 1999), as they provide a foundation for our socio-emotional development and general understanding of others, from cradle to grave. During adolescence (11–18 years), the first romantic relationships emerge (Connolly et al., 2014). Romantic relationships are commonly defined as “ongoing voluntary interactions that are mutually acknowledged which have a peculiar intensity and the intensity can be marked by expressions of affection – including physical ones and, perhaps, the expectation of sexual relations, eventually if not now” (Collins, 2003, p. 2). Being in a romantic relationship can contribute to the well-being of youth but it can also have negative consequences (Gómez-López et al., 2019; Kansky et al., 2018). Youth involved in a positive romantic relationship have greater life satisfaction, higher self-esteem, and fewer mental and physical health problems (Davila et al., 2017; Gómez-López et al., 2019). Aspects associated with a positive romantic relationship include commitment, intimacy, support, good communication, effective decision-making, and healthy conflict management (Gómez-López et al., 2019). On the other hand, an unhealthy romantic relationship, including aspects such as power imbalance, hostile behavior or dating violence, is associated with poor mental health, such as higher levels of depression and anxiety (Gómez-López et al., 2019; Kansky et al., 2018). In addition, the quality of and experiences in the first romantic relationships of youth shape romantic relationships later in life (Collins et al., 2009; Kansky et al., 2019). Therefore, it is important to further examine the positive and negative aspects of the romantic relationships of youth as they provide building blocks for youth’ later family functioning.

Romantic relationships of youth are at least partly shaped by experiences during childhood (Collins et al., 2009; Wolfe et al., 2004). One review found that youth who grow up in a stable and harmonious family context are more likely to experience pleasant romantic relationships, whereas youth who grow up in a context of unsafety (e.g., due to family violence) are more likely to experience unpleasant romantic relationships, for example because dating violence is an integral part of their romantic relationship (Collins et al., 2009). The current study will focus specifically on the romantic relationships of youth with a history of family violence.

Two important theoretical frameworks providing an explanation for the behavior and feelings of youth in a romantic relationship are attachment theory (Bowlby, 1969; Cassidy & Shaver, 2016) and observational learning theory (Burgess & Akers, 1966). First, from the perspective of attachment theory, beliefs and expectations about self and others are guided by an individual’s (internal) working model, which is based on previous (early) interactions between children and their caregivers (George, 1996; Muller et al., 2012). A secure attachment between the child and caregiver is important for a healthy development of information processing and emotion regulation (Van der Kolk, 2005). Partly through early interactions with their caregivers, securely attached children have learned to give words and meaning to their feelings (i.e., they are able to ‘mentalize’; cf. Bateman & Fonagy, 2013) and to adapt in challenging situations, whereas insecure children often have feelings of mistrust and insecurity about others and themselves. They also tend to misinterpret situations and are therefore more likely to react defensively or aggressively (George, 1996; Muller et al., 2012). Second, from the perspective of observational learning theory, people develop positive or negative beliefs or attitudes about aggressive and violent behavior through the learning processes of observation and imitation (Burgess & Akers, 1966). Moreover, these learning processes are important for the development of emotion regulation (Huesmann et al., 2011). By observing interactions between family members, children learn how to respond to situations, but also how to understand and manage their own emotions as well as how to interpret the emotions of others. Based on these theories, youth exposed to family violence may have different reactions to, and expectations of, a romantic partner.

Several studies show that youth exposed to family violence struggle with various aspects of romantic relationships. For example, youth exposed to family violence may find it difficult to take the perspective of their romantic partner or they may not feel competent to handle complicated and sensitive issues (Wolfe et al., 1998, 2004). Furthermore, due to past experiences they are more likely to experience feelings of fear, mistrust and hostility in their romantic relationship as they constantly expect to be rejected by their romantic partner (Connolly et al., 2014; Van der Kolk, 2005). Moreover, due to insecure or anxious attachment, and feelings of anger, they are more likely to control their partner, which may lead to aggressive and violent behavior (Connolly et al., 2014; Follingstad et al., 2002). In addition, the literature clearly shows that youth exposed to family violence have an increased risk of becoming either a victim or perpetrator of dating violence (Dardis et al., 2015; Kaukinen, 2014; Vagi et al., 2013), also known as the intergenerational transmission of violence (Montalvo-Liendo et al., 2015; Smith-Marek et al., 2015; Stith et al., 2000). Thus, youth exposed to family violence may experience challenges in their romantic relationship. Our study aims to explore these challenges.

Most studies on challenges within the romantic relationships of youth exposed to family violence have used a quantitative approach and often do not include the experiences of youth themselves. Asking about the experiences of youth, which is done in qualitative research, is important to provide nuanced information about a mechanism or phenomenon. A qualitative study on how early adolescents (10–13 years) exposed to violence between their parents make sense of a romantic relationship showed that these young adults describe a romantic relationship as healthy when partners experience love and joy, do activities together, support each other, and are able to communicate effectively, whereas they describe a romantic relationship as unhealthy when there is anger, distrust or abuse (Richardson et al., 2021). Furthermore, a qualitative study on dating violence in romantic relationships of young adults (12–19 years) with a disrupted childhood (such as experiencing family violence) revealed that they had little experience with having a positive romantic relationship, which they described as including aspects such as love and commitment, trust, and good conflict resolution (Wood et al., 2010). Furthermore, this study revealed that youth who experienced family violence strongly felt they did not want to repeat the violence, however some also perceived this as nearly impossible. Moreover, the young women in particular reported having low self-esteem as a result of previous family violence. This low self-esteem was also associated with experiences of acceptance of dating violence in their romantic relationships. Finally, some qualitative studies reveal that youth link a history of family violence during childhood to experiencing dating violence in a romantic relationship (Edwards et al., 2016; Lavoie et al., 2000; Reed et al., 2008). Notwithstanding these valuable insights, what is currently lacking is a more holistic perspective on the romantic relationships of youth with a history of family violence. For this reason, more qualitative research on the experiences within the romantic relationships of youth exposed to family violence is necessary (Richardson et al., 2021).

Although there are a few qualitative studies that include both experiences within romantic relationships of youth and a history of family violence, these studies have not extensively explored cognition, affect and behavior of youth exposed to family violence. It is expected that youth exposed to family violence will experience challenges in their romantic relationships. However, the literature also indicates that some youth exposed to family violence succeed in breaking the intergenerational transmission of violence and also learn new ways of relating to a romantic partner (Renner & Slack, 2006; Richardson et al., 2021; Suzuki et al., 2008). So it is also important to explore positive experiences within the romantic relationships.

The main goal of our study is to qualitatively explore both challenges and positive experiences within the romantic relationships of youth exposed to family violence, and the perceived role of previous family violence. In-depth interviews with youth aged between 16 and 20 years are conducted. All youth were exposed to family violence during childhood, which is defined in the present study as both direct child abuse (violence from a caretaker to the youngster) and indirect child abuse (witnessing intimate partner violence). Specifically, the current qualitative study explored:

1. What challenges do youth exposed to family violence during childhood experience within their romantic relationships during adolescence?
2. What do these youth think and feel about the link between being exposed to family violence during their childhood and the challenges they subsequently face within their romantic relationships during adolescence?
3. What are the positive experiences of these youth within their romantic relationships, and what evidence is found for their resilience over time?

More information about how youth who experienced family violence think about and react to their romantic relationships, and their perceptions of how their attitudes, feelings and behaviors are shaped by their (violent) upbringing, may contribute to future prevention of dating violence, and possibly also other aspects of intergenerational violence. By qualitatively exploring these questions, new hypotheses can be generated about the possible impact of family violence on dating violence and other negative experiences of youth within their romantic relationships. Furthermore, we will be able to examine what kind of tools can be given to youth who have been exposed to family violence, but also to inform practitioners who work with these children, adolescents, and young adults, to decrease the risk of negative experiences and to increase positive experiences within their romantic relationships.

## Method

The present study was part of the larger longitudinal research project “Violence within the home and its impact on parents’ and children’s lives”. (Steketee et al., 2021). We specifically used data from semi-structured interviews with 16-20-year old youth who already participated in this larger longitudinal study.

### Recruitment and Participants

For the larger longitudinal study, families who experienced family violence were recruited, via convenience sampling, through child protection services in the Netherlands (Steketee et al., 2021). The procedure is described into more detail in earlier studies (Lünnemann et al., 2019, 2022). To recruit participants for the present study, purposive sampling was used. Therefore, youth aged 16 years or older who participated in the longitudinal study were approached by email (*n* = 126). The email contained information about the study and our inclusion criteria: youth with a minimum age of 16 years and having (had) a romantic relationship for at least 3 months. However, we only got three responses to this email. Therefore, if a phone number was known, youth were also called to ask if they were willing to participate. Eventually, nineteen youth participated. However, one participant was excluded after conducting the interview because during her interview it became clear that she had not experienced any family violence during her youth.

Our final sample consisted of ten women and eight men aged between 16 and 20 years (M = 18 years). All eighteen interviewees were given pseudonyms and their characteristics are presented in Table 1. Being in a romantic relationship was self-defined. On average, interviewees were 14 years old when they had their first romantic relationship. During the interview, eight interviewees reported that they were currently in a romantic relationship. All interviewees had been engaged in romantic relationships with someone of the opposite sex, but one interviewee mentioned that he was also interested in people of the same sex. Five of the interviewees told that there were other boyfriends or girlfriends in between these romantic relationships, but they themselves did not indicate that as a serious romantic relationship because they were too short. The romantic partners of boys on average were somewhat younger than the interviewee (range − 1 to 0 years), whereas girls on average had a romantic partner that was older (range − 1 to 5 years).

**Table 1:** Characteristics of the interviewees

Pseudonym	Sex	Age	Currently in a romantic relationship	Number of romantic relationships (including current)
Tess	Female	19	Yes	2
Rohan	Male	20	No	3
Liam	Male	17	No	2
Nick	Male	18	No	1
Maud	Female	17	Yes	2
Liz	Female	17	Yes	2
Anjali	Female	19	Yes	3
Amir	Male	17	No	2
Jaylinn	Female	19	Yes	2
Julian	Male	19	No	3
Nathalie	Female	19	Yes	5
Hannah	Female	17	No	2
Malia	Female	18	Yes	6
Jim	Male	19	No	2
Lucine	Female	18	No	2
Sem	Male	19	No	2
Milo	Male	16	Yes	2
Eva	Female	16	No	2

### Procedure

The data consists of semi-structured interviews with youth. All interviews were conducted by the first author and a research assistant. The location of the interview was chosen by the participants, with as condition that no other persons were present in the room where the interview was conducted. This was important to ensure the participant was able to talk freely and confidentially. The first six interviews were conducted at the participants’ home. Due to COVID-19 restrictions, the other twelve participants were interviewed via online video calls using Microsoft Teams.

The goal of this study was to describe the narratives about the perception and experiences on the romantic relationships of youth who experienced family violence during their youth. A guideline was designed with the topics that had to be covered and several questions that could be asked. Open-ended questions were used to stimulate storytelling, and these questions were followed by additional questions. We started the interview with a relationship matrix to summarize the characteristics of all romantic relationships in chronological order (cf. Van de Bongardt & Verbeek, 2021). For each romantic relationship we asked the length, age of respondent, age and sex of romantic partner, and a number between one and ten to indicate their satisfaction with a particular relationship. They also specified their most important relationship. First, we asked the questions about this most important romantic relationship. After each (sub)topic we asked if they had different experiences in their other romantic relationships. First, we asked youth about (un)healthy aspects of a romantic relationship in general, and next positive and negative aspects of their own romantic relationships. Furthermore, the topics understanding/empathy, talking about emotions, trust, conflicts and dating violence were addressed by all youth, but other topics could be addressed by youth also. Moreover, to gain insight into the role of family violence experienced in childhood on the romantic relationships of youth reflective questions are asked, such as “*why do you think you react(ed) this way in your romantic relationship*” or “*how do you reflect on arguing with your romantic partner like that at that time*”.

Furthermore, we used the method of photo elicitation, which means using a photograph to evoke feelings, memories, and information (Glaw et al., 2017). Participants were asked beforehand to search on the internet for two images; one image that reflects their childhood and one image that reflects their romantic relationship(s). Searching images stimulated participant to think about important characteristics of their youth and romantic relationships in advance. Respondents started with an explanation of these images. This encouraged the interviewee to talk about their own experiences and perspectives of their romantic relationships and their youth.

Interviews were recorded and manually transcribed verbatim. Transcripts were anonymized. The recording of one interview failed, therefore we only had the notes of this interview. The duration of interviews ranged from 41 to 92 min (M = 67). Participants were compensated for their time with €15.

Both beforehand and during the interview attention was paid to ethical aspects. At least one week prior to the appointment, participants were given written information about the purpose of the study, confidentiality and the possibility of not answering a question or withdrawing at any time without consequence. This information was given again at the beginning of the interview, after which informed consent was signed for participation of the study as well as for recording of the interview. During the interview, attention was paid to the well-being of the participant. At the end of each interview the participant was asked how they had experienced the interview. If participants needed help, contact details of different organizations were given as well as the possibility to talk to a licensed psychotherapist, who was also a member of our research team. This study was approved by the Ethics review Committee of the Erasmus University Rotterdam (20-012).

### Analysis

The verbatim transcripts of the interviews were uploaded into ATLAS.ti software (version 9) for coding and data analysis. Coding, analyzing, and writing up the findings was an iterative and recursive process done by the first author in collaboration with a reflective team consisting of the other authors.

First, we focused on the manifest content of the interviews, which is the literal text that is visible (Kondracki et al., 2002). In line with theoretical thematic analysis (Braun & Clarke, 2006) we first coded the interviews using a code list created by the first author in consultation with the other authors. This code list was based on the topics derived from theory and previous studies (e.g., trust, understanding each other, dating violence). Next, in line with indicative thematic analysis, open coding was used (Braun & Clarke, 2006). So, the code list was expanded with codes of other negative and positive experiences raised by youth about their romantic relationships, such as support, and that conflicts were never solved. We also coded the narratives of youth in which they linked the experiences within their romantic relationships to their (violent) upbringing themselves, such as being accustomed to screaming and swearing. The codes emerged from these steps were close to the narratives. Afterwards, codes with a similar meaning were merged.

Next, we expanded the indicative thematic analysis with looking at the latent content; the underlying ideas, assumptions, and conceptualizations of the text (Braun & Clarke, 2006). Therefore, the interviews were read and re-read to look for narratives in which challenges, the role of experiences of family violence during childhood, and signs of resilience were talked about in a more implicit way. For example, codes that emerged from this analysis were: putting oneself in the shoes of a romantic partner, clinging to a romantic partner, flight as reaction to conflict, low self-esteem.

The second level of analysis consisted of searching for themes, reviewing these themes and defining and naming the themes (Braun & Clarke, 2006). Also, illustrative quotations were selected and discussed in the reflective team to ensure that each quotation was a good representation of that theme.

## Findings

### Challenges in the Romantic Relationships of Youth

Challenges that emerged in the interviews with youth exposed to family violence about their romantic relationships were: lack of support and appreciation, finding a balance between connectedness and autonomy, setting boundaries, difficulty trusting others, regulating emotions, and conflict management.

#### Lack of Support and Appreciation

A major challenge youth spoke of is the giving and receiving of support and appreciation in their romantic relationships. The youth also stated that they find support and appreciation to be very important in a romantic relationship, but at the same time it appeared from their stories that they did not feel supported and appreciated in at least one of their romantic relationships. The lack of support stemmed from a feeling that they couldn’t open up to their romantic partner, they didn’t feel like they were genuinely interested in each other, or that they didn’t feel taken seriously by their romantic partner. For example, Maud said: “*Yeah, sometimes he just seems to see it all as a joke. I do think that sometimes. Like he thinks; ‘no worries, it’s all good’ or something like that, even though I feel like it is a serious issue*”. However, several youth stated that their romantic partner was also unable to understand them, Rohan explained “*I don’t feel understood anyway [laughs] (…) It’s just because…well. Sometimes I don’t even fully understand myself*”. According to some youth, this was because they were in a situation or experienced things that are incomprehensible to others. Besides that, it also appeared that youngsters have difficulty providing the right support to their romantic partner, for example because they have difficulty putting themselves in their romantic partner’s shoes, like Milo “*Or things that other people worry about, which others can fully understand but I can’t, and I’ll just be thinking ‘what are they complaining about?‘ for example*”.

The lack of appreciation with some of the youth was because they did not feel valued as a person, as was the case with Rohan “*It is a bit difficult to explain, but I didn’t feel a sense of appreciation in that relationship (…) I didn’t feel as though I was taken into consideration as much as I should have been*”. Other youth felt that their romantic partner did not appreciate what they did for him or her in the romantic relationship, including Hannah “*I am naturally quite caring myself. I tend to dive in headfirst and want to help people and I’ll often just say ‘Oh let me do that for you’ […] at a certain point they start to take that for granted*”. One aspect associated with giving and receiving support and appreciation is how connected youth feel with their romantic partners, a challenge we describe in the next section.

#### Finding a Balance Between Connectedness and Autonomy

A second challenge that youth face is the search for balance between connectedness and autonomy. They find it important to have a healthy balance between being connected to their romantic partner and having a life of their own and being independent. At the same time, youth have difficulty with this distance versus closeness in their romantic relationships. They seem to either find themselves on one end of the spectrum (highly focused on independence) or the other (highly focused on closeness). For example, there are youth who had trouble committing to their romantic partner. They found it difficult to be open with their romantic partner, cut themselves off or end the romantic relationship as soon as it gets serious. Other youth have trouble letting go of their romantic partner, by constantly justifying their romantic partner’s (negative) behavior. Or they even cling to their romantic partner (or each other), like Malia “*all you want … at least in my case … you just want to see each other every single day. (…) We’re just miserable if we don’t see each other. (…) I don’t know why, but we just get cranky with each other. We’ll tell each other repeatedly ‘we miss each other so much right?‘. And then we just agree ‘yes, so much’. [Laughs]*”. For these youth the emphasis is on the connection with their romantic partner, which may indicate that they are afraid of losing the other person. Some youth also mentioned this specifically, like Jim “*And at a certain point I just let everything slide, just because I didn’t want to lose her*”. They do everything they can to not lose their romantic partner, which also touches on the next challenge: how do you set your own boundaries in your relationship?

#### Setting Boundaries

A third challenge in the romantic relationships of youth is setting boundaries. We know that youth exposed to family violence have often been exposed and pushed beyond their limits. This is reflected in their romantic relationships. Some youth said they didn’t dare to go against their partner when they didn’t agree with something, didn’t dare to be honest with their partner, or they often took the blame when the partner got angry with them. Other youth said they would change their personality, including Rohan “*I tend to change my own personality for the other person. (…) This would include things like asking her what she wanted in a partner*” or doing everything for the other person, like Liz “*I had to pay for everything for him and if he did have money then he’d just spend it on cigarettes or drugs and besides that he’d let me pay for everything and take care of the housekeeping… I also had to do everything around the house, from cooking dinners to laundry and cleaning so yeah…*”. It was difficult for these youth to recognize their limits and to indicate them. Several youth described the negative feeling this gave them. Tess said that she found it suffocating “*Yes that was quite suffocating to be honest, because you feel constantly stressed, you are not allowed to do this and that, even though you want to*”, and Malia agreed “*Yeah, that drove me crazy mentally too. I was really just giving all of myself in that relationship and that really exhausted me mentally*”. Or youth lose their self-esteem because they overstep their boundaries, like Anjali “*I lost my self-worth because of him to be honest (…) It just broke me because I constantly would wonder what I was doing wrong? What am I doing wrong? Why always me, why does everything seem to be my fault?*“.

#### Difficulty Trusting Others

A fourth aspect that many youth find important, but also struggle with in their romantic relationship, is trust. Half of the youth themselves indicated that there was no (mutual) trust in at least one of their romantic relationships. Several youth said that they themselves had trouble with trust, especially when it comes to romantic partners being open and honest with them. Maud had the following to say about this: “*for example, the beginning of this relationship I’m in now, I found [trust] difficult because he was hanging out with friends who smoke weed every day (…) I asked him to quit for me and he did. And he stopped doing it since then, but yes in the beginning of course this was difficult because you know that he is addicted to it, so it is very difficult to be able to say, I trust you’*”. Several youth also spoke of distrust on the part of their romantic partner, which they noticed in the form of jealousy. Liam commented on the topic “*Because she [romantic partner] was really jealous. I had this one friend, we didn’t really hang out much, I just saw her from time to time. It was just casual, like ‘hi, how’s it going’. But she [romantic partner] just got really jealous*”.

However, the other youth were ambiguous about trust in their romantic relationships. On the one hand, these youth said that there was trust in their romantic relationship, but they also mentioned aspects that appear to reveal mistrust. Malia spoke about this “*Yes, he trusts me completely. Yeah. He always says it. He’ll repeatedly say, ‘I trust you completely’. But at the same time, he does get quite jealous. He really does. He always asks, ‘where are you, who are you with’. I don’t mind answering that at all, because I do the same*”. The statements made by youth also showed that they had trouble trusting that their romantic partner would not leave them. Distrust from the romantic partner, and sometimes also from the youth themselves, manifests itself in many romantic relationships in the form of controlling behavior, as was the case with Sem: “*If I left to go to town or something, I had to say with who, where, at what time*”. A number of youth also said that their controlling behavior was necessary and therefore justified in order to gain confidence in their romantic partner, like Amir: “*I regularly checked her phone [without her knowing] to see if she no longer had contact with certain exes and she didn’t, so in the end I just left it and realized I can trust this girl*”.

*Youth with a jealous or controlling romantic partner described this behavior often as annoying, they became stressed and thought it was unjustified. Jaylinn said the following about this: “Yes, that was quite annoying (…) I usually got angry about it… Then I’ll think to myself ‘please, just a little bit of trust would be nice sometimes’… I’ve never done anything weird or anything”. One respondent said that a lack of control can be very scary because you don’t know exactly where your romantic partner is, like Malia “suffocating. I didn’t know what he was doing, who he was with. I would wonder ‘what is he getting up to.‘ That’s it basically. It’s just a very suffocating and frustrating feeling”.*

#### Difficulty Regulating Emotions

Another challenge youth face in their romantic relationship is expressing and regulating their emotions. For example, youth said they could not talk about emotions with their romantic partner, such as Rohan “*We hardly talked about emotions now that I think about it. She was also very closed off about it. (.) Every time I brought it up, she would suddenly have something she needed to do or whatever, you know, or the conversation would just end abruptly. Or it would be with short, one-word answers*”. As Jim would put it “*Well, I dunno. I never do this (…) When I was angry I would say: ‘Leave me alone.’ If I was sad it would be: ‘Leave me alone.’ If I was agitated: ‘Leave me alone’*”.

Even the youth who were able to talk about emotions with their romantic partner, had difficulty regulating their emotions. Liam explained “*Now that I’m getting older, my fuse is also getting shorter, I notice I’m getting angry quicker. (…) when I’m angry, I can’t control myself*”. Youth find that they can easily get annoyed or irritable and find it difficult to deal with this or put it into words with their romantic partner. As a result, youth often bottle up their emotions, causing them to overflow at a certain point, to burst, as the quote from Julian shows “*I also would bottle up my emotions, and they would all come out in one go. It just became so many mixed feelings. So it wasn’t one emotion, but multiple emotions*”. Several youth also said that they can quickly react abruptly or emotionally, Nick gave an example: “*For example after… If someone makes a joke I can take it very literally and then a switch flips in me and then I just get angry*”. It seems that youth tend to interpret things that their romantic partner says or does in a negative way and, as a result, they overreact or are easily irritated.

#### Limited Conflict Resolution Skills

A final challenge in the romantic relationship of youth is not letting conflicts escalate. Despite the fact that youth called an unhealthy aspect of a romantic relationship having a lot of intense conflict, almost all youth also said that in at least one romantic relationship they often argued with their romantic partner. Some youth even described their romantic relationship as toxic, when we asked what Anjali meant by this, she explained “*Just that we argue all the time and then we are together, things happen and then things are good again for a while and then the arguing just starts again*”. Almost all of these youth also said that they shout at each other during these arguments. In addition, it turned out that more than half of the youth had to deal with psychological or physical violence, as Tess’ quote shows: “*Then he grabs my arm, for example, because he wants to keep me there, but I want to get out so … and there’s nothing else I can do, because I can’t remove my arm or whatever, so I just hit him on the arm or, well, I’m not sure where else him hit*”. Youth commented that they called each other names and would say hurtful things, that they were beaten or punched, or that they fought with each other. Some youth also said that there was sometimes a power struggle, Malia said: “*He had quite a lot of power over me (…) In every sense. And I mean it. He sort of kept me as a slave. That’s the way to look at it. I did everything for him*”. Other youth would speak in more discrete terms, like, their romantic partner was very forceful or controlling in the romantic relationship. As a result, youth conform to their partner or their freedom is restricted. For example, Liam explained: “*I was also not allowed to talk to friends, especially female friends, but also male friends*”.

During or after the conflict, many youth have anger management issues. As a result, they react in a primal way as though under imminent threat and enter fight or flight mode, or freeze. Most youth withdraw when faced with a conflict with their romantic partner, they leave and ignore their romantic partner. Amir, for example: “*I would stop talking to her or just get up or, you know, just leave. I didn’t feel like being with her anymore, not even for a moment*”. We could describe the remaining youth as the pursuers, like Eva. She said: “*I’d really start to yell at him and he would get really angry too, but he could usually walk away from the situation. And often I thought this was a good thing, but sometimes I just didn’t agree with that approach and then I would get even angrier*”.

Most of the youth indicated that they didn’t resolve the issue by talking about the arguments (when they saw each other again), they continued as if it hadn’t happened, or they talked about it but nothing really changed and the fighting continued. Hannah explained: “*I could discuss it, but it didn’t really sink in… Then I just realized he wasn’t really… He heard what I was saying, but he didn’t listen. He… It just didn’t seem to register and then he would do it again the next day*”. In addition, some youth said that their anger only came out after they left each other, as Jim’s quote shows “*[the fuse] it would always blow after she left. (…) Then the door would suffer the consequences, or the wall, or the bottles of perfume. It would always be something… you need to release the anger. You’re not going to take it out on your 1.60-meter-tall girlfriend. Obviously that’s not the way to go about things*”. These youth need to release their anger and aggression and because they don’t want to hit their partner, they prefer to destroy an object.

### Insecurity in Youth and Challenges in Romantic Relationships

In previous sections, we discussed challenges youth exposed to family violence face in their romantic relationships. Research shows that the challenges youth experience in their romantic relationship can be traced back to the problems they experienced in their childhood (Connolly et al., 2014; Follingstad et al., 2002; Van der Kolk, 2005; Wolfe et al. al., 1998, 2004; Wood et al., 2010). Below we take a closer look at the connection between youth’s childhood experience and the challenges in their romantic relationship.

The challenge youth face in accepting support and appreciation in a romantic relationship may be because they didn’t receive positive feedback about themselves in their interaction with caregivers and therefore have not developed a healthy sense of self-esteem. Jaylinn explains: “*My stepfather was not the nicest person at that time (…) There was a lot of swearing and telling how worthless I was at the time and [clears throat] … that I’d be better off leaving*”. Many youth feel that they are unworthy. Because of this, they may look for validation from their romantic partner that they are indeed worthy of being there and find support and appreciation very important.

The difficulty in forming a healthy bond in their relationship can thus arise from the unhealthy relationship the youth have with their parents. Nathalie commented about the relationship with her father: “*He was very protective and nurturing. He continued to do this as I got older, he tried very hard to shelter and protect me. Because of this we got into fights […] I wanted to go camping when I was 14, when I packed my things and said I was going, I wasn’t allowed to leave him. But I went anyway. Then the police brought me back, after he had called them. Then I was taken home, but then I said he couldn’t stop me and I left again*”. It is possible that dysfunctional relationships cause youth to have difficulty finding the right balance between connectedness and autonomy in their current relationship. Youth indicate that they are afraid to let go and lose their romantic partner. That makes them go beyond their own boundaries to keep their romantic partner happy.

Furthermore, youth indicated that they find it difficult to be open about their feelings and problems in their romantic relationship and to trust their romantic partner will not abandon them. This may be due to the feeling they are on their own in life, as Sem also indicates “*Don’t feel sorry for yourself, just live your life, because later on it’s like ‘you’re on your own anyway, so to speak’*”. This feeling could be related to their feeling that their parent(s) did not support them and that they could not turn to their parent(s) with their emotions and problems.

In addition, a challenge for youth is that they are easily irritable or have difficulty regulating their emotions. Because of this, they tended to react more fiercely in their romantic relationship than they would like, or they get into arguments with their romantic partner more quickly. Several youth linked this to the insecurity of their youth. When we asked Liam where his pent-up emotions came from, he said, “*Yes, my mother’s ex was an alcoholic. He sometimes became very aggressive towards me and my brother. […] He also… he hit me and my brother and then, well let’s just say it would escalate. […] you’d hear that screaming over and over and I actually didn’t feel safe at home anymore*”. Due to negative experiences at home, youth are wary and feel hypervigilant, but also do not know what to do with those feelings causing them to bottle up their emotions. Youth have seen themselves changed by the fights at home, they said they have become tougher and still have a lot of anger in them. For example, Jim says “*I was always a very quiet kid. (…) And then with the fights between my parents, then I started standing up to my dad. Then I started to realize that when I got angry I actually had a lot of physical strength. That’s where I got the hang of it. Then I got to see a side of myself that I really wish I hadn’t. I also had a period when I didn’t go out because I was afraid of myself. That I might lose my temper at someone*”. The pent-up feelings and anger they experience can effect on how youth react in their romantic relationship: they may suddenly react with anger or aggression. Fights, which are part of every relationship, evoke strong emotions in youth such as anger, but also fear or the need to avoid conflict: they run away instead of attempting to talk things out.

Furthermore, more than half of the youth related the screaming and aggressive behavior during a conflict in their romantic relationship to the violence they experienced in their youth. Youth said they are used to yelling and swearing and that they don’t care if their romantic partner does that. Hannah explains: “*Well, yeah it doesn’t bother me that much, because that’s what I was used to at home. So yeah… I’ve built up a bit of a wall against that, so that doesn’t bother me. (…) [her father] he screams loudly and… (…) every sentence he uses the word ‘fuck’ at least 20 times. So all the swearing and the… I’m used to it*”. Some of the youth also said that they knew deep down that it is not good to react in this way in a conflict, but that it comes naturally, as was the case with Rohan: “*It has become inevitable. I really don’t want to, but it always ends up happening. I just end up screaming, involuntarily, without even noticing*”.

Finally, the interviews revealed that youth struggle with the necessary competences (skills) when it comes to solving problems and conflicts in relationships. Youth say that they didn’t learn at home how to enter into a dialog or resolve an argument in a normal way, such as Nathalie “*We [Nathalie and her romantic partner] never manage to resolve things and I think because I don’t know how to solve problems, because nobody ever set a good example. No one ever taught me how to solve things in a normal way*”. Arguments were not resolved in their childhood, the problem was ignored by (step) parents and confrontation was avoided.

### Positive Experiences and Learning Capacity

In addition to the challenges mentioned above, several interviews showed that despite their negative childhood experiences, youth also have positive experiences and learn from new experiences in their romantic relationships. As a result, several youth spoke about positive experiences, mainly in their current or most recent romantic relationship.

The romantic partners of the youth, often current or most recent, play an important role in the positive and corrective experiences of these youth. For example, there are youth who indicated that they felt supported and appreciated by their romantic partner: they could express themselves, their romantic partner was there for them when they felt stressed or sad, listened and tried to understand them and this gave them comfort. Milo explains: “*Well, just for example, if I tell her something, she then asks me questions about it. She shows that she is listening to you, considering what you’re saying*”. The accounts of the youth also showed that if they had a romantic partner who was good at communicating and sharing his or her emotions, youth also noticed that they were more open and could talk about emotions. For example, Milo continued about his current romantic relationship: “*Yes, I’m able to express myself with her. I can talk about anything. It’s never an issue at all. (…) when I’m sad she puts an arm around me and then we just… we talk it out*”. Some youth also mentioned that they managed to resolve the conflicts they had in the right way, Eva said: “*We just talked things out and we both would usually end up apologizing. And we discussed the subject and then well, for example, we would look at how things went wrong in the first place*”. It seems that this is because youth get a romantic partner that they can be with and learn from.

Not only can the youth be pushed by and learn from their romantic partner, but parents-in-law can also play an important role in teaching how to resolve conflicts in a good way. Just to give an example, Sem’s parents-in-law talked to him and his girlfriend after an argument they had had, during which Sem actually wanted to walk out the door “*There was a conversation around the table, even with the parents, who would sit with them and listen carefully and they would just give us tips, I’m quite grateful to them for that, because then we manage to talk things out together again*”.

There are also youth who have had therapy (professional support) which makes it possible for them to talk about problems with others, including their romantic partner. Jaylinn indicates “W*hen I went into therapy … I think it’s then that I learned to deal with myself in a better way (…) To talk about things. I think so. I think my therapy has improved my communication with other people a bit yes*”.

These accounts show that youth can escape the bad experiences at home by using their ability to learn and then apply this in a romantic relationship, gaining new positive experiences and breaking the cycle of violence and trauma.

## Discussion

The current study explored challenges and resiliency of youth exposed to family violence in their romantic relationships and the perceived role of previous family violence in childhood. The findings of the current study expand on earlier studies about the intergenerational transmission of violence by drawing on narratives of youth aged between 16 and 20 years, investigating a holistic perspective of both challenges and positive experiences in the romantic relationships of youth and the link to their childhood.

Similar to the findings of previous studies (e.g., Connolly et al., 2014; Follingstad et al., 2002; Van der Kolk, 2005; Wolfe et al., 1998, 2004; Wood et al., 2010), our study reveals that youth exposed to family violence experience challenges in their romantic relationship. Youth reported a lack of support and appreciation from their romantic partner. At the same time, youth sometimes found it difficult to support and trust their romantic partner. In addition, according to the narratives, youth struggle to find the right balance between connectedness and autonomy. On the other hand, youth also described how they often let others overstep their personal boundaries, causing stress and negative feelings about themselves. Youth narratives also indicated several aspects of mistrust within their romantic relationships, which was often associated with controlling behavior by either their romantic partner or themselves. In addition, youth discussed finding it difficult to talk about their feelings. Youth narratives indicate that they struggle with regulating their emotions. Finally, most youth talked about having serious conflicts with their romantic partner, and described finding it difficult to resolve their problems, indicating that they have limited conflict resolution skills.

Although these challenges are normal for youth to experience in their romantic relationships, our findings show that youth exposed to family violence experience multiple challenges simultaneously, and their responses are related to their insecurities during their youth. Youth exposed to family violence often interpret their romantic partner’s behavior often more negatively and are sensitive to rejection (Connolly et al., 2014). In addition, youth exposed to family violence often act out of a stress response, making it harder to store new information and learn from mistakes (Davies et al., 2016). This makes it more difficult to learn and grow, whereas youth who have not experienced violence grow with their partner on these issues by falling down, getting up and continuing to talk about their problems.

Half of the youth in this study reported being a victim or perpetrator of psychological or physical dating violence in their romantic relationship. This is consisted with several studies concluding that dating violence is prevalent in youth romantic relationships (Black et al., 2010; Offenhauer et al., 2011; Wincetak et al., 2017), especially among youth exposed to family violence (Dardis et al., 2015; Kaukinen, 2014; Vagi et al., 2013). Moreover, several studies found factors related to dating violence similar to challenges arising of our study; inability to cope with stress, insecurity, and the lack of knowledge about healthy communication in a relationship were related to experiencing dating violence (Edwards et al., 2016; Spencer et al., 2021). This suggests that if these challenges are not addressed, the youth in our study who did not experience dating violence are at increased risk of experiencing dating violence in the future.

The present study expands on previous studies by asking youth exposed to family violence about all their challenges within romantic relationships instead of focusing on a specific challenge. An important finding is that, although the challenges are described as separate themes, there seems to be a strong relation between the different challenges. Due to a lack of support, trusting oneself and others and communication, youth report that they feel less connected to their romantic partner, lose confidence in their romantic partner, give in to demands to avoid conflict and in doing so increasingly let others cross their boundaries. According to the interviewees, this leads to conflicts, which are often not resolved, resulting in even more (and more serious) conflicts, and in some cases also in dating violence.

The link between experiencing family violence during childhood and being a victim or perpetrator of dating violence is well established (Dardis et al., 2015; Edwards et al., 2016; Kaukinen, 2014; Lavoie et al., 2000; Reed et al., 2008; Vagi et al., 2013). The findings of the present study add to previous studies that the narratives of youth also reveal that family violence youth experience during their childhood contributes to the challenges they face in romantic relationships. Due to an unsafe environment when growing up, youth often have low self-esteem and a distorted relationship with their parents, resulting in difficulties trusting oneself and others, as well as with connecting in a healthy way with their romantic partner. Furthermore, youth talked about not having learned how to regulate their emotions or how to resolve conflicts from their (step)parents. Also, youth have become accustomed to the violence, while at the same time they still experience a lot of anger and are hypervigilant due to the experienced family violence. Consequently, in their romantic relationships youth suppress their emotions, more often misinterpreted situations, conflicts become worse and problems are not worked out.

Both attachment theory (Bowlby, 1969; Cassidy & Shaver, 2016) and observational learning theory (Burgess & Akers, 1966) may explain why youth exposed to family violence will experience challenges within their romantic relationship. Youth explained the perceived role of previous family violence especially on the level of behavior. They talked about not being able to observe what a healthy romantic relationship should look like, giving support for the observational learning theory. Interviewees described they had not learned how to talk about problems; problems in their family of origin were ignored rather than solved, and that they were accustomed to (psychological) violence because it happened all the time at home during childhood. Interviewees linked this to the challenges they experience within their romantic relationships; their inability to problem solve often resulted in loss of control over emotions and behaviors in arguments with their romantic partner. On the other hand, interviewees also told they would not accept violence and did not think of violence as normal behavior in a romantic relationship. This indicates that, in contrast to what is described in observational learning theory, they do not have a positive belief or attitude to the use of violence.

Whereas explanations of youth themselves for the relation between the challenges in their romantic relationships and previous family violence are in line with observational theory, the more implicit findings in the narratives of youth are also consistent with mechanisms described in attachment theory, which focuses more on cognitions and affect. As described in attachment theory, (internal) working models, encompassing beliefs and expectations about self and others, are based on (early) interactions between children and caregivers (George, 1996; Muller et al., 2012). The findings of the current study reveal a link between interactions in childhood experiences of youths and their difficulty in trusting oneself and their romantic partner. Some youth gave their trust too easily to a romantic partner who abused their trust, while other youth seemed to be very reluctant to trust others. This may indicate a sense that they do not need others and are emotionally self-sufficient. From an attachment perspective, this is best understood as reflecting not only how youth view others; are the people around me generally trustworthy? But also how youth view themselves; am I able to comfort myself in times of distress or do I need others, especially for emotional support and reassurance. Youth who have more self-confidence, are often better able to regulate their emotions. This makes them less dependent on others for emotional reassurance and enables them to make more selective distinctions between those who can be trusted and those who cannot.

The narratives also imply a link between childhood experiences of youth and their struggle with finding the right balance between being connected to their romantic partner on the one hand and being autonomous on the other hand. Most of the youth in our study find themselves on the extremes of this continuum. First, the narratives of youth strongly focused on connection with their romantic partner show a lack of trust in themselves; youth do not have the feeling they are valuable, and they need affirmation from their romantic partner to feel good about themselves. There are also feelings of mistrust as they fear of being left by their romantic partner. Consequently, they are constantly looking for confirmation and cling to their romantic partner or justify their aggressive behavior. Several interviewees also spoke about a romantic partner who was fixed on the connection with them, often resulting in controlling behavior. For some interviewees this made them feel safe; they were less afraid of abandonment by their romantic partner.

Second, the narratives of youth strongly focused on autonomy showed that they do not talk about their problems, and suppress negative feelings and emotions. Also, youth spoke about still feeling a lot of anger due to their childhood and being hypervigilant, consequently, they sometimes reacted more aggressively because they misinterpreted the situation. These youth usually described avoiding the confrontation; they left when tension built between them and their romantic partner or when they had an argument. About their childhood, youth described they had the feeling of being on their own in life and therefore have learned to deal with problems on their own, indicating that youth only trust on themselves and find it difficult to have faith in their romantic partner.

Third, some of the youth spoke about both aspects, being strongly connected to their romantic partner and yet being very autonomous. These findings suggest that the youth included in our study possibly have an insecure attachment (Cassidy & Shaver, 2016) due to previous interactions with caregivers, which might have contributed to the challenges they faced within their romantic relationship. This is supported by previous research that concluded that children exposed to family violence are more likely to have an insecure attachment with caregivers (Baer & Martinez, 2006; Sousa et al., 2011). Moreover, several aspects of how youth feel, think and react in their romantic relationship are recognized belonging to the different insecure attachment styles described by Ainsworth: anxious-ambivalent, avoidant, and disorganized (Collins & Read, 1990). However, the current study did not measure these different attachment styles. Further research is required to examine this hypothesis by investigating the relation between the challenges found in our study and the different attachment styles of youth exposed to family violence, using an instrument to measure attachment styles of youth.

We may conclude that the experiences of family violence during childhood have their repercussions on the experiences of youth within their romantic relationships. The relation between challenges youth face within their romantic relationships and the previous family violence can be explained on the level of cognition, affect and behavior. Therefore, findings of the current study give support to observational learning theory as well as attachment theory. While both theories are widely accepted, recent studies have also criticized the lack of inclusiveness of these theories. For example, studies argue that attachment theory was primarily developed in a Western context, but that sensitive responsiveness and warmth are not universal (Keller et al., 2018; Morelli et al., 2018). The main criticism of social learning theory is that this theory does not take into account the influence of genetic factors, and that this theory assumes that communities and our learning environments are stable, whereas the world is constantly changing (Stewart, 2021). Future research on romantic relationships of youth exposed to family violence should pay attention to both mechanisms, but also bear in mind that these theories might not be inclusive.

Finally, an important finding of the current study is that youth exposed to family violence also talked about positive experiences within their romantic relationships. Youth described they felt supported and appreciated by their romantic partner, they learned to talk about their problems and feelings, and sometimes they also learned to solve their conflicts. From the interviews it became clear that these positive experiences mainly occurred in their current or most recent romantic relationship. In addition, youth talked about other learning experiences such as parents-in-law who acted as a role model or youth had received professional support where they learnt communication skills they could apply in their romantic relationship. The finding that youth exposed to family violence are able to learn and have positive experiences within their romantic relationships is in line with previous research showing that youth exposed to family violence are resilient and both social and professional support can buffer the effect of previous family violence (Holt et al., 2008). Moreover, supporting the literature on attachment theory (Sroufe, 2005), these positive and learning experiences also show that (internal) working models are changeable, and road blocks can be transformed into building blocks.

### Strengths, Limitations and Directions for Future Work

This qualitative study adds to the existing literature on how childhood exposure to family violence relates to youth’s cognition, affect and behavior in their romantic relationships. Despite the challenge in reaching our specific participant group, we conducted eighteen interviews that lasted on average more than one hour. Therefore, we had rich and detailed material to analyze. Most participants found the interview helpful as it gave them a voice, and some felt relief because it was one of the first times they told their story. This aligns with other studies highlighting the unintentional therapeutic aspects of interviews (Lowes & Gill, 2006), underscoring the importance of conducting qualitative studies by maltreated youth.

While our study contributes valuable insights, some limitations should be considered. Firstly, due to time constraints, we primarily focused on the most significant romantic relationships of our interviewees, potentially missing important aspects of their other romantic relationships. However, youth learn from each romantic relationship they have been engaged in (Smith et al., 2010). Therefore, future research that explores each romantic relationship in depth might illuminate this learning process. Secondly, our sample consisted of youngsters in opposite sex relationships identifying themselves as either male or female. Therefore, our findings may not be generalized to romantic relationships of LBGTQ youth. Thirdly, the present study did not analyze gender differences. However, the literature indicates that gender differences are important when investigating the impact of family violence as well as investigating dating violence in the romantic relationships of youth (Barter & Stanley, 2016; Wood et al., 2010). Future research should consider gender differences when examining the impact of family violence on the youth’s romantic relationships.

Our findings confirm that experiences during childhood are important for later experiences during the romantic relationships of youth, at least from their perspective. While our study gathered one partner’s perspective, romantic relationships involve interactions between two individuals. The findings of the current study show that youth exposed to family violence can learn from their romantic partner, overcoming some challenges they experienced in previous romantic relationships. On the other hand, if both romantic partners grew up in an unsafe family context it is more likely that problems will arise in the romantic relationship, and that these problems eventually may lead to dating violence, although it is impossible to make any causal claims on the basis of our current findings. Some quotes used in our study also indicate problems in the family context of the romantic partner. It is therefore essential to consider both partners’ childhood experiences to better understand the mechanisms of the intergenerational transmission of violence.

Additionally, significant changes in romantic relationships, like cohabitation, may be a trigger for the development or escalation of problems and violence, especially for those who struggle to cope adaptively with stressors (Nieder & Seiffge-Krenke, 2001; Shulman & Connolly, 2013). Our findings suggest that youth exposed to family violence often find it difficult to deal with problems in an adaptive way, making them vulnerable during transitions like moving in together or starting a family. In addition, several youth talked about letting off steam by destroying things when they or their romantic partner had left. However, when living together it is less easy to go somewhere else. This makes it crucial to follow up on the romantic relationships of the youngsters of the current study.

### Implications

The findings of the current study shed light on possible mechanisms that explain the intergenerational transmission of dating violence. Knowledge on challenges and resilience in the romantic relationships of youth and the role of previous family violence, may inform the work of practitioners working with youth exposed to family violence (e.g., social workers, psychologists). This study highlights the importance of providing youth exposed to family violence with communication and conflict resolution skills, help to improve their self-esteem and self-confidence and to pay special attention to the impact of their childhood experiences and interactions with caregivers. Furthermore, it is important to ask youth about the challenges in their romantic relationship, as well as positive experiences and their (social) support system. Internal working models are partly formed in early childhood through interactions between children and their parents. However, internal working models can be reformed later in life through other social relationships and experiences, such as in romantic relationships or in-laws. An early focus on challenges, resilience and the learning ability of youth might prevent youth exposed to family violence to become a victim or perpetrator of dating violence.

Overall, this qualitative study presents that youth exposed to family violence struggle with multiple aspects of their romantic relationships (e.g., trust, conflict resolution) and these challenges are intertwined. In their narratives, youth link these challenges to their childhood experiences (e.g., family violence, not learning to solve problems). However, they also display resiliency and show that having support from a romantic partner, family-in-law, or a supportive professional gives them the opportunity to deal with these challenges in an adaptive way, and are able to have positive experiences in their (future) romantic relationships. If indeed youth with the help of those around them are able to transform roadblocks into building blocks, this likely has huge positive implications for the wellbeing of future generations.
